# Color M-Mode Echocardiography for Non-Invasive Assessment of the Intraventricular Pressure in Dogs Before and After Ductus Arteriosus Occlusion: A Retrospective Study

**DOI:** 10.3389/fvets.2022.908829

**Published:** 2022-07-12

**Authors:** Miki Hirose, Ahmed S. Mandour, Seijirow Goya, Lina Hamabe, Katsuhiro Matsuura, Tomohiko Yoshida, Momoko Watanabe, Kazumi Shimada, Akiko Uemura, Ken Takahashi, Ryou Tanaka

**Affiliations:** ^1^Department of Veterinary Surgery, Tokyo University of Agriculture and Technology, Tokyo, Japan; ^2^Department of Animal Medicine (Internal Medicine), Faculty of Veterinary Medicine, Suez Canal University, Ismailia, Egypt; ^3^Department of Bioresource Sciences, Nihon University, Fujisawa, Japan; ^4^Division of Veterinary Research, Department of Veterinary Surgery, Obihiro University of Agriculture and Veterinary Medicine, Obihiro, Japan; ^5^Department of Pediatrics and Adolescent Medicine, Juntendo University Graduate School of Medicine, Bunkyo, Japan

**Keywords:** dog, ductus arteriosus, Doppler echocardiography, intraventricular pressure, diastole, overloading

## Abstract

**Background:**

Novel non-invasive evaluation of the intraventricular pressure differences and gradients (IVPD and IVPG) by color M-mode echocardiography (CMME) is a promising method in diastolic function evaluation. Patent ductus arteriosus (PDA) is a congenital heart defect which is associated with increased preload. The present work provides a clinical trial for the assessment of IVPD and IVPG changes in dogs before and after surgical occlusion of PDA.

**Materials and Methods:**

A total of 12 client-owned dogs were enrolled in this study. PDA was confirmed using echocardiography, and all dogs underwent PDA occlusion. Conventional echocardiography and CMME were conducted on each patient on the operation day (Pre-PDA) and 48 h after its occlusion (Post-PDA). The total IVPD and total IVPG, as well as segmental intraventricular pressure (basal, mid-to-apical, mid, and apical) were measured from Euler's equation using specific software (MATLAB). Data were analyzed for variability and for the difference between pre- and post-PDA. The effect of PDA occlusion on the measured variables was calculated using biserial ranked correlation (rc).

**Results:**

There was a significant reduction in end-diastolic volume, fraction shortening, stroke volume, and mitral inflow velocities (early and late) after PDA closure. CMME was feasible in all dogs, and the CMME indices showed moderate variability, except for the apical segment of IVPD and IVPG. After PDA closure, in comparison with the pre-PDA occlusion, there was a significant reduction in total IVPD (2.285 ± 0.374 vs. 1.748 ± 0.436 mmHg; *P* = 0.014), basal IVPD (1.177 ± 0.538 vs. 0.696 ± 0.144 mmHg; *P* = 0.012), total IVPG (1.141 ± 0.246 vs. 0.933 ± 0.208 mmHg; *P* = 0.032), and basal IVPG (0.578 ± 0.199 vs. 0.377 ± 0.113 mmHg; *P* = 0.001); meanwhile, mid, mid-to-apical, and apical segments of both IVPD and IVPG showed non-significant difference. The magnitude of PDA occlusion on the measured variables was clinically relevant and associated with a large effect size on total and basal IVPD and IVPG (rc > 0.6).

**Conclusion:**

The current clinical study revealed matched response of IVPD and IVPG to the reduced preload rather than left ventricular relaxation. This result is an initial step in the clinical utility of CMME-derived IVPD and IVPG measurements in the diastolic function evaluation in dogs with PDA that warrants further clinical studies.

## Introduction

During the fetal life, the vascular tunnel, known as “ductus arteriosus,” shunts the blood between the aorta and the main pulmonary artery to bypass the immature nonfunctional fetal lungs. At birth, the abrupt increase in the blood oxygen content and the reduction in the blood prostaglandin levels stimulate the closure of the duct. In dogs, functional closure of the ductus occurs 4 days post-partum and becomes complete at 7–8 days of age (1–3). However, hypoplasia and asymmetry of the ductal architecture result in patent ductus arteriosus (PDA), which is the most frequently reported congenital cardiac defect that affects a wide range of dog breeds. PDA is accounted for 20–30% of the congenital heart defects in dogs depending on geographical screening (4). PDA is routinely diagnosed on clinical examination with echocardiography which shows volume overloading (5, 6). Depending on the size of the PDA, the clinical signs of PDA are variable ranging from no or mild symptoms to severe congestive heart failure signs including dyspnea, continuous heart murmurs, abnormal pulses, and exercise intolerance. The PDA in dogs can be managed medically or occluded surgically through a transvenous catheter or *via* thoracotomy. Rapid interference for surgical occlusion of the PDA tunnel is preferable before overt clinical symptoms and irreversible heart damage (2).

Recently, CMME-derived intraventricular pressure differences (IVPD) and intraventricular pressure gradients (IVPG) have been invented as promising tools for cardiac function examination. Studies revealed the utility of IVPD and/or IVPG for the diagnosis of diastolic dysfunction. The feasibility of the novel approach has been utilized in various experimental animal models (7–13), as well as in some human studies (14–16). Recent studies showed that non-invasive IVPG could be an indicator of heart failure in children as well as in dogs receiving chemotherapy (12, 15). IVPG also explained the mechanism underlying diastolic dysfunction in the tetralogy of Fallot, which was associated with reduced suction force as indicated by reduced mid-to-apical IVPG (14). Another study suggested that IVPD and IVPG indices could reflect the preloading conditions (9). However, more studies are still needed for the validation of IVPD/IVPG in the clinical setting.

Current cardiovascular research focuses on the development of non-invasive diagnostics for diastolic dysfunction. This is because heart failure with preserved ejection fraction (HFpEF) is known to account for half of the patients diagnosed with end-stage heart failure, and the accurate diagnostic methods are challenging and have not yet been established (17). Evaluation of diastolic function using conventional echocardiography is limited as the measurements are affected by the heart rate and arrhythmias. A combination of various echocardiographic techniques is necessary to avoid overestimation of the echocardiographic measurements (18). Additionally, the practical usage of a reliable cardiac catheterization method is invasive and requires perioperative procedures, including anesthesia, and surgical techniques (19, 20). To our knowledge, there are no reports of clinical studies which have focused on the importance of IVPD and IVPG analysis obtained from CMME in canine congenital heart disease. PDA is a good example of volume overloading, which resulted in a profound left to right shunt from the aorta to the pulmonary artery leading to left side dilatation, increased end-diastolic pressure, myocardial failure, and overt pulmonary edema (6, 21). We hypothesize that the hemodynamic and contractility changes which occur before and after PDA closure could be detected by the measurement of IVPD and IVPG using CMME. The present study aims to provide a short-term evaluation of IVPD/IVPG changes in dogs with PDA.

## Materials and Methods

### Animals and Study Protocol

A total of 12 client-owned dogs echocardiographically diagnosed with PDA and underwent transvascular PDA occlusion were enrolled in this study. The study was carried out between January 2018 and April 2021 at the Animal Medical Center, Tokyo University of Agriculture and Technology, Japan. Before enrollment, the informed consent was collected from all owners.

### Clinical Examination and Diagnosis of PDA

Diagnosis and decision for the surgical treatment of PDA were determined through a comprehensive clinical evaluation including detailed medical history, physical examination (including cardiac auscultation, mucous membrane color, breathing condition, etc.), as well as thoracic radiography, electrocardiography, and echocardiography. On cardiac auscultation, grade IV continuous left basilar systolic murmur was detected in all cases. Specific objects/parameters to be evaluated before surgery were as follows: signalment (age, breed, sex, and body weight), anatomical characterization of PDA, existence and severity of heart failure, as well as systolic and/or diastolic dysfunction.

### PDA Closure

Patent ductus arteriosus occlusion was performed *via* intercostal thoracotomy under general anesthesia. All preoperative, intraoperative, and postoperative procedures were conducted as described in previous reports (22–24). All dogs were sedated with buprenorphine hydrochloride (Lepetan; Otsuka Pharmaceutical Co., Ltd., Tokyo, Japan, 0.02 mg/kg, intravenously), midazolam hydrochloride (Dormicum; Astellas Pharma Inc., Tokyo, Japan, 0.2 mg/kg, intravenously), and atropine sulfate (Atropine sulfate; Tanabe Seiyaku Co., Ltd., Saitama, Japan, 25 μg/kg, intravenously). After tracheal intubation, anesthesia was induced with propofol (Propofol Mylan; Mylan Seiyaku, Tokyo, Japan, 4 mg/kg, intravenously) and was maintained by isoflurane inhalation (Isoflurane for Animal Use; Intervet, Osaka, Japan, end-tidal concentration of 1.5 ± 0.1%). The ductus arteriosus was occluded using a double hemostatic clip (DS titanium ligation clip, Aesculap, Japan). During surgery, transesophageal echocardiography was performed with ProSound SSD-α10 (Hitachi Aloka Medical, Ltd., Japan) using a sector probe (UST-52119S, Hitachi Aloka Medical, Ltd., Japan). Postoperative confirmation of hemostatic clip and leakage was assessed by Doppler echocardiography.

### Conventional Echocardiography

Standard echocardiography was performed on all unsedated dogs at the baseline on the operation day before surgical occlusion of the PDA (Pre-PDA) and 48 h after its occlusion (Post-PDA). The echocardiography was performed using ALOKA prosound α10 (Hitachi Aloka Medical, Ltd., Japan) with a 5-MHz phased array transducer probe (UST52108, Hitachi Aloka Medical, Ltd., Japan). Dogs were placed in lateral recumbency and the conventional echocardiographic examination from the standard right and left cardiac windows was recorded using a well-known standard echocardiographic technique (25). The following measurements were reported from the right parasternal short-axis view at the papillary muscle level: left ventricle (LV) end-diastolic and end-systolic diameters (LVIDd, LVIDs), interventricular septal thickness in diastole and systole (IVSd, IVSs), LV free wall thickness in diastole and systole (LVPWd, LVPWs), LV end-diastolic and end-systolic volume (EDV, ESV), and fraction shortening (FS). From the right parasternal short-axis view at the base of the heart in late systole, the left atrium diameter (LADs), aortic diameter (Ao), and the left atrial diameter to aortic diameter ratio (LA/Ao) were obtained by two-dimensional echocardiography. Also, the right ventricular outflow tract (RVOT) was measured by pulsed-wave Doppler echocardiography of the main pulmonary artery. On the left apical view, the left ventricular outflow tract (LVOT) and mitral inflow velocity indices (early velocity, Ev; late velocity, Av) were measured using pulsed-wave Doppler echocardiography, while tissue Doppler imaging (TDI) was used to measure the annular tissue velocity indices. The TDI measurements include systolic velocity (Sm) as well as early and late diastolic tissue velocities (Em and Am) from IVS (septal, Sep) and LVPW (free wall, FW). The corresponding ratio between Em/Am, as well as E and Em (E/Em) from IVS and LVPW was then calculated.

### Color M-Mode Echocardiography for Assessment of IVPD and IVPG

The whole echocardiography protocol including CMME was performed within 15 min in which the CMME was traced within 5 min. To avoid the stress-related erroneous imaging, the examination was temporarily stopped when there was an abrupt increase in respiratory and heart rates. The ultrasound probe was set to trace IVPG from the left apical two-chamber view with proper visualization of the entire mitral inflow tract to the LV apex. To increase the Nyquist limit, a prior machine setting including sweep speed of 300 mm/s and color baseline shift was adjusted at −64. During image processing for IVPD and IVPG using the MATLAB, the time from aortic valve opening to its closure, the time from the beginning of Q wave to the start of early mitral inflow (E wave), and the time from the start of Q wave to the peak E wave were collected from conventional echocardiography and manually inserted into the dialog boxes of the MATLAB. Five high-quality images of consecutive heartbeats were selected and analyzed separately by the same observer, and the average measurement of each patient was considered for the analysis.

The IVPD and IVPG derived from CMME were calculated based on the previously validated IVPD measurements technique (14, 26, 27). IVPD was calculated from the Euler equation after processing images obtained *via* the CMME using MATLAB (The MathWorks, Natick, MA, USA) as follows:

(∂ P)/(∂s) = –ρ((∂v)/(∂ t) + v (∂v)/(∂ s)).

where ∂ is the change in element followed, P is the pressure, ρ is the constant blood density (1,060 kg/m^3^), v is the velocity, s is the position along with the color M-mode line, and t is the time. The IVPG values were derived from the IVPD according to the following formula (14, 26–28):

IVPG (mmHg/cm) = IVPD/LV length.

The IVPG and IVPD were automatically divided into basal, mid-to-apical, mid, and apical segments, which are corresponding to their location in the LV (11, 12, 14).

### Statistical Analysis

The sample size of dogs before and after the closure of PDA was measured based on the outcomes and calculations performed with the G^*^Power 3.1.9.2 software (University Kiel, Germany, 1992–2014) (29) assuming a moderate effect of PDA occlusion on CMME variables according to Cohen with 0.37 effect size (30). The normality of the data was tested by the Shapiro–Wilk test and the 95% coefficient of variations (CV) and 95% confidence interval (CI) of means were reported. Wilcoxon matched-pairs signed-rank test was used to compare all echocardiographic data before and after PDA occlusion using GraphPad Prism Version 8 (GraphPad Software Inc., San Diego, CA, USA). Analyzed variables showing *P* < 0.05 were considered statistically significant. Spearman's rank correlation was measured between conventional cardiac parameters and CMME indices. To examine the effect of PDA closure on the obtained parameters, the effect size for non-parametric data was calculated by rank-biserial correlation (rc) using a free JASP software program. Interpretation of the effect size was done based on rc values as follows (31): small effect (rc ≥ 0.1), medium effect (rc ≥ 0.4), and large effect (rc ≥ 0.6).

## Results

### Clinical Findings and Outcomes

Twelve small breed dogs underwent surgical treatment for PDA. Five dog breeds were included in this study: Pomeranians (33.3%, *n* = 4), Chihuahua (25%, *n* = 3), Maltese (16.7%, *n* = 2), Mix (16.6%, *n* = 2), and Toy Poodles (8.3%, *n* = 1). Overall, seven males (58.3%) and 5 females (41.7%) were included. On the day of diagnosis, the median BW was 1.49 kg (range: 0.92–2.16) and the median age was 4.5 months (range: 2–7). One dog had diarrhea and was prescribed anti-diarrhea medicine and antibiotics by the referring clinic before surgery. Also, one dog showed cough and another one had polyuria and polydipsia. No dog showed severe symptoms of heart failure or received other medication.

### Conventional Echocardiography

**Figure 1:**
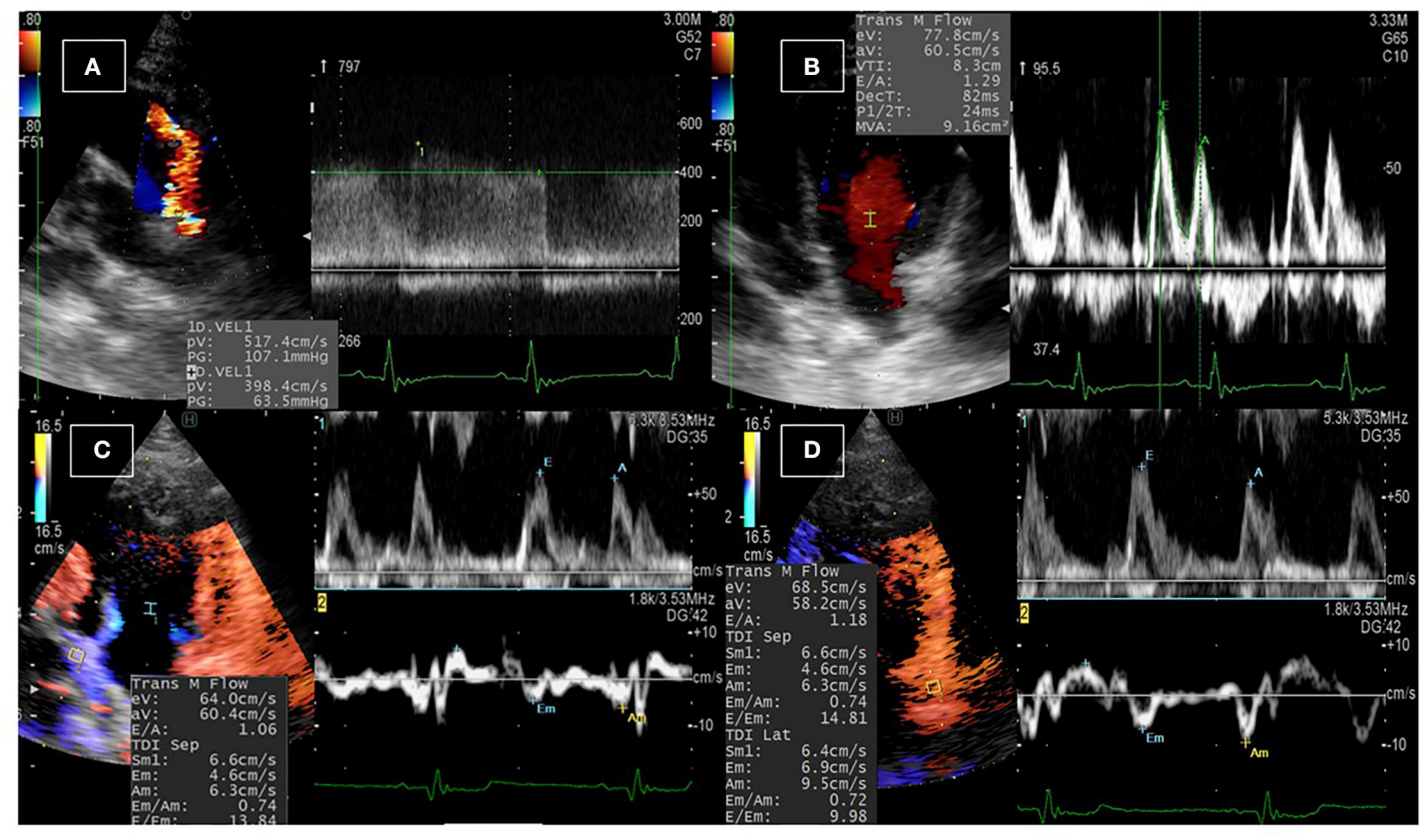
Conventional echocardiographic examination in dogs with PDA. **(A)** The PDA shunt was detected upon echocardiographic examination from the left parasternal short-axis view at the base of the heart using color Doppler echocardiography, and the velocity of the blood flow across the shunt was detected using continuous-wave Doppler echocardiography. **(B)** Pulsed-wave Doppler echocardiography of the transmitral flow was used to measure the early and late diastolic peaks. **(C,D)** Dual Doppler imaging of the transmitral flow and the tissue Doppler imaging at the septal **(C)** and the free **(D)** wall of the left ventricle to measure the early inflow velocity to early tissue velocity (E/Em) ratio.

Figure 1 illustrates the conventional echocardiographic findings in dogs diagnosed with PDA. The left–right PDA shunt was detected from the left cranial parasternal location after obtaining the heart base in the short axis. Table 1 shows functional measurements of conventional echocardiography. The data revealed a significant increase in IVSd and LVPWd thickness, and a significant reduction in LVIDd, heart rate, EDV, FS, and mitral inflow waves (Ev and Av). By TDI, Sm of the septum and the E/Em of the free wall were significantly reduced. The effect size on the aforementioned heart function parameters was large (rc > 0.6). Before the operation, the median of PDA velocity was 532.3 cm/s (range: 436.3–594.9 cm/s). The following abnormalities were observed in the postoperative settings: mitral regurgitation (MR) was found in eight dogs (median MR velocities: 571.5 cm/s, dp/dt: 3,123.7 mmHg/s); pulmonary regurgitation (PR) in four dogs (median PR: 146.0 cm/s); and tricuspid regurgitation (TR) in one dog (TR velocity: 169.6 cm/s). All dogs were discharged from the hospital 48 h after showing hemodynamic stability.

**Table 1:** Echocardiographic measurements in dogs before and after PDA occlusion.

**Variables**	**Pre-PDA**	**Post-PDA**	***P-*value**	**Effect size**
IVSd	3.90 (2.50–4.40)	4.55 (3.70–5.100	0.007	−0.879
LVIDd	22.65 (16.40–32.20)	19.55 (14.20–25.20)	0.001	1.000
LVPWd	3.35 (3.00–4.80)	4.05 (3.30–7.60)	0.006	−0.927
IVSs	5.70 (4.10–12.10)	6.45 (4.10–10.00)	0.331	−0.364
LVISd	13.00 (9.50–20.10)	12.60 (8.30–18.50)	0.266	0.394
LVPWs	6.35 (4.40–7.60)	6.70 (5.30–7.90)	0.100	−0.561
HR	150.0 (103-166.00)	130.0 (66.0–162.0)	0.014	0.818
EDV	11.5 (4.40–33.00)	7.50 (2.90–16.00)	0.001	1.000
ESV	2.2 (0.80–8.10)	2.0 (0.60–6.40)	0.528	0.218
FS%	42.2 (28.00–55.50)	34.7 (20.90–57.20)	0.034	0.692
LADs	12.65 (1.60–18.40)	11.0 (6.30–16.70)	0.096	0.485
Ao	9.10 (1.20–10.90)	9.2 (7.90–10.40)	0.783	0.145
LA/Ao	1.44 (1.01–1.75)	1.10 (0.79–1.74)	0.106	0.455
RVOT	7.70 (7.50–7.90)	8.6 (4.70–11.90)	0.002	0.621
LVOT	9.15 (5.70–10.90)	9.05 (4.20–11.70)	0.835	−0.127
Ev	97.70 (77.80–124.50)	74.90 (51.40–104.50)	0.001	1.000
Av	67.85 (58.10–89.80)	43.35 (32.30–68.10)	0.001	1.000
Sm Sep	7.90 (4.40-9.50)	6.60 (3.20–9.50)	0.040	0.667
Em Sep	8.70 (6.30–12.60)	6.90 (4.50–10.60)	0.066	0.603
Em/Am Sep	1.35 (1.04–2.22)	1.18 (0.59–2.34)	0.424	0.182
E/Em Sep	10.77 (8.10–19.02)	10.20 (8.84–13.79)	0.077	0.515
S FW	7.90 (5.00–10.70)	7.25 (3.70–9.90)	0.436	0.269
Em FW	12.90 (5.70–17.80)	11.25(7.10–16.90)	0.226	0.410
Em/Am FW	2.0 (0.99–8.98)	1.95 (1.49–3.25)	0.470	−0.242
E/Em FW	7.28 (5.31–17.53)	5.54 (4.20–7.41)	0.003	0.879

*Values are presented as median (data range). The comparison was done between the obtained echocardiographic measurements in the same individuals before and after PDA occlusion. P < 0.05 was considered statistically significant*.

*rc, ranked biserial correlation. LVIDd, LV end-diastolic diameters; LVIDs, LV end-systolic diameters; IVSd, interventricular septal thickness in diastole; IVSs, interventricular septal thickness in systole; LVPWd, LV free wall thickness in diastole; LVPWs, LV free wall thickness in systole; EDV, end-diastolic volume; ESV, end-systolic volume; EF, ejection fraction; FS, fractional shortening; LA/Ao, left atrial diameter to aortic diameter ratio; RVOT, right ventricular outflow tract; LVOT, left ventricular outflow tract; HR, heart rate; Ev, the early diastolic velocity of mitral inflow; Av, the late diastolic velocity of mitral inflow; E/A early to late mitral inflow ratio; S, systolic annular velocity, Em, early tissue annular tissue velocity; Am, late annular tissue velocity; Em/Am, early to late annular tissue velocity ratio. Sep, septal wall; FW, free wall*.

*E/Em early mitral inflow to early tissue velocity ratio*.

### Color M-Mode Echocardiography

**Figure 2:**
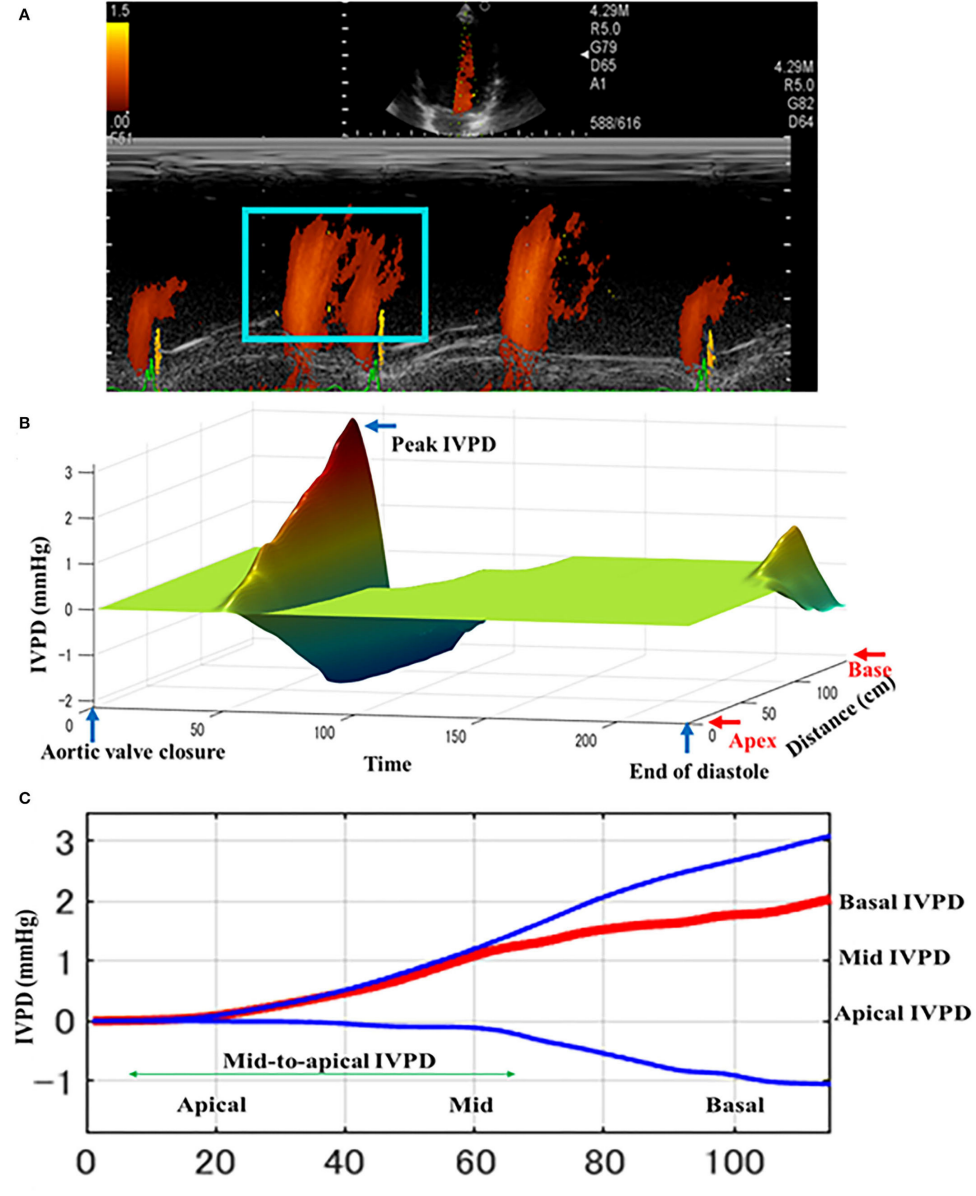
Schematic illustration of intraventricular difference (IVPD) measured by Color m-mode echocardiography (CMME) in dogs before PDA occlusion. The mitral inflow was firstly optimized from the left apical four-chamber view. After that, the machine setting for CMME to measure IVPD (IVPG) was switched on and photos were captured **(A)**. Offline saved photos were further processed by MATLAB software for IVPD and IVPG calculation. The three-dimensional profile of IVPD was calculated after extraction of the velocity, temporal, and spatial elements from the region of interest (blue box) **(B)**. Spatial distribution of the IVPD along the entire left ventricle from the base toward the apex **(C)**. The top (blue), middle (red), and bottom (blue) lines represent inertial, total, and convective IVPD, respectively.

The CMME was successfully performed in all dogs (Figure 2). All dogs showed standard spectral mitral inflow waves of CMME with the opening and closure of the mitral valve. The obtained IVPD and IVPG results from MATLAB yielded five different indices, total, basal, mid-to-apical, mid, and apical. Table 2 shows the descriptive statistics of CMME variables in dogs before and after PDA occlusion. All CMME data were normally distributed, except mid-to-apical IVPD. The variability of CMME data from pre- and post-PDA was separately calculated, which showed a comparable 95% CV. All IVPD and IVPG indices obtained from pre- and post-PDA showed moderate variability (95% CV <25%), except for the apical IVPD and apical IVPG (95% CV = 36.9 and 44.8, respectively). The variability of IVPD and IVPG data between heart cycles was not significant.

**Table 2:** Variability and normality of CMME indices before and after PDA occlusion.

**Variables**	**Pre-PDA**			**Post-PDA**		
	**Mean ±SD**	**95% CI (L-U)**	**95% CV**	**Mean ±SD**	**95% CI (L-U)**	**95% CV**
Total IVPD	2.21 ± 0.37	1.82–2.61	16.8	1.74 ± 0.29	1.82–2.61	16.89
Basal IVPD	1.14 ± 0.24	0.80–1.47	21.3	0.72 ± 0.16	0.80–1.47	21.63
Mid-to-apical IVPD	1.13 ± 0.24	0.90–1.35	21.3	1.00 ± 0.20	0.90–1.35	20.40
Mid IVPD	0.91 ± 0.17	0.75–1.08	18.8	0.77 ± 0.15	0.75–1.08	18.96
Apical IVPD	0.19 ± 0.07	0.09–0.29	36.9	0.25 ± 0.08	0.09–0.29	32.72
Total IVPG	1.12 ± 0.21	0.94–1.29	18.5	0.94 ± 0.18	0.94–1.29	18.90
Basal IVPG	0.54 ± 0.10	0.42–0.67	18.8	0.39 ± 0.08	0.42–0.67	21.34
Mid-to-apical IVPG	0.56 ± 0.11	0.46–0.66	19.1	0.57 ± 0.11	0.46–0.66	19.89
Mid IVPG	0.47 ± 0.10	0.40–0.54	20.8	0.43 ± 0.08	0.40–0.54	19.24
Apical IVPG	0.099 ± 0.044	0.044–0.155	44.8	0.133 ± 0.059	0.044–0.155	44.6

*The intraventricular pressure difference (IVPD) and intraventricular pressure gradients (IVPG) indices before and after occlusion of the patent ductus arteriosus (PDA) in dogs (n = 12)*.

*CV, coefficient of variation; CI, confidence interval (lower and upper limit)*.

### Effect of PDA Closure on IVPD and IVPG Measurements

**Figure 3:**
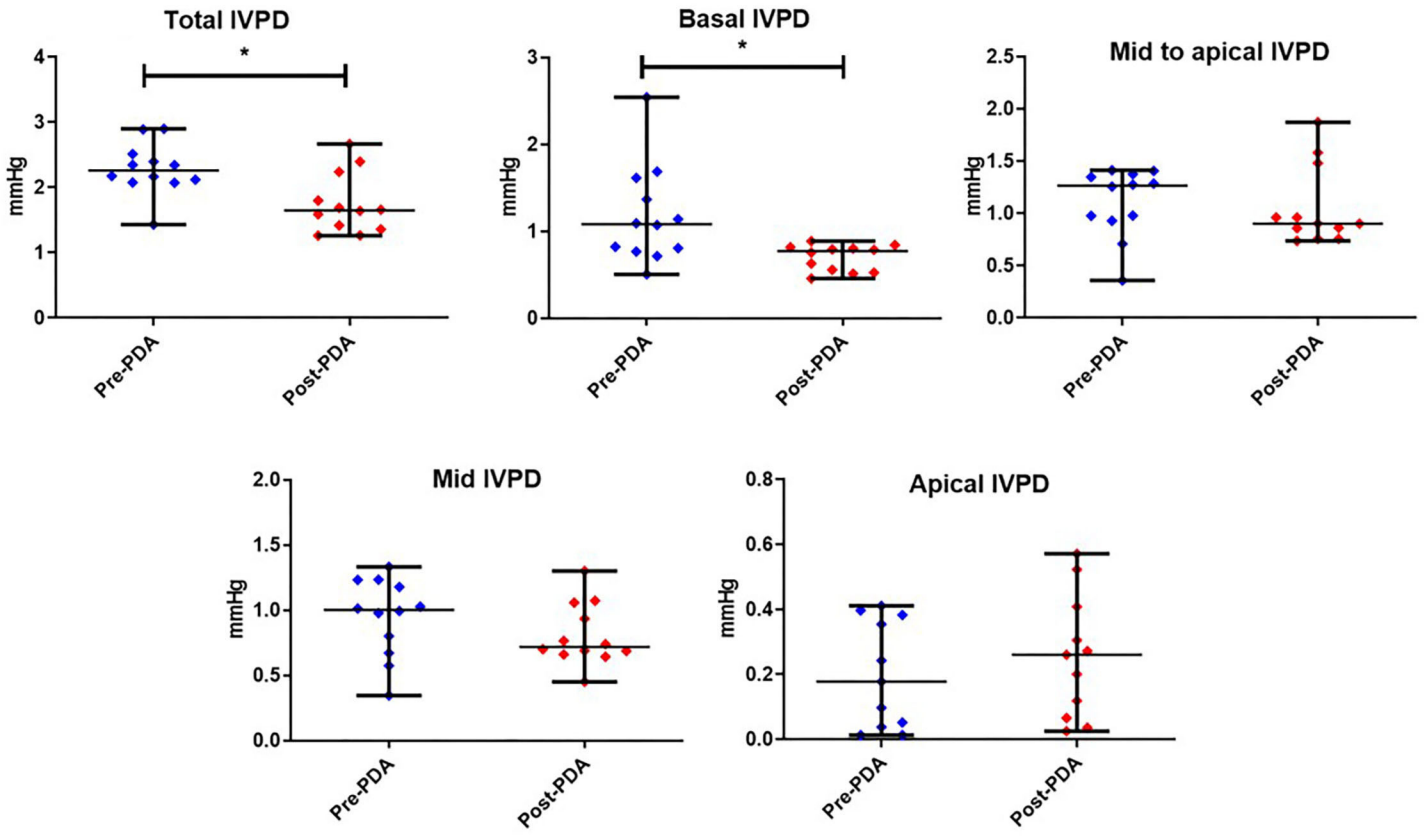
CMME-derived intraventricular pressure difference (IVPD) variables before and after occlusion of the patent ductus arteriosus (pre-PDA, post-PDA) in dogs (*n* = 12). Dots plots showing the median and range. *indicates the statistical significance (*P* < 0.05).

**Figure 4:**
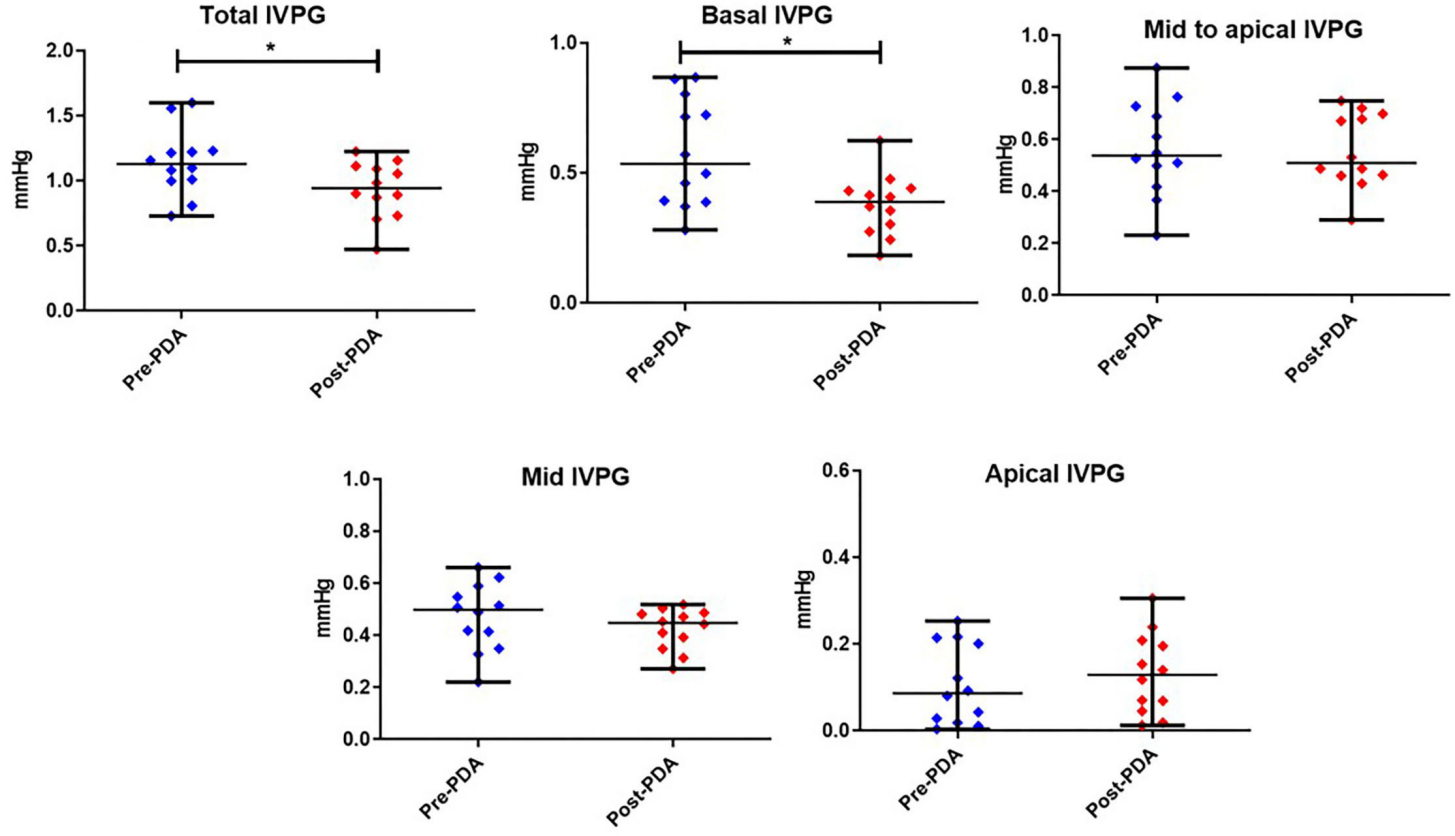
Dots plots showing the median and range of the intraventricular pressure gradients (IVPG) indices Pre-and Post-PDA closure in dogs (*n* = 12). *reveals the statistical significance (*P* < 0.05).

Figures 3, 4 represent the results of the IVPD and IVPG indices before and after PDA occlusion. The data revealed a significant reduction in total IVPD (Mean ± SD: 2.285 ± 0.373 vs. 1.748 ± 0.436 mmHg; *P* = 0.014), basal IVPD (1.177 ± 0.538 vs. 0.696 ± 0.144 mmHg; *P* = 0.012), total IVPG (1.141 ± 0.246 vs. 0.933 ± 0.208 mmHg; *P* = 0.032), and basal IVPG (0.578 ± 0.199 vs. 0.377 ± 0.113 mmHg; *P* = 0.001) after PDA closure. The percentage of reduction in total IVPD, basal IVPD, total IVPG and basal IVPG after PDA closure were 23.5% (0.537 mmHg), 40.9% (0.481 mmHg), 18.23% (0.208 mmHg), and 34.8% (0.201 mmHg), respectively. In addition, there were non-significant increases in apical IVPD (0.198 ± 0.157 vs. 0.253 ± 0.180 mmHg) and apical IVPG (0.107 ± 0.088 vs. 0.131 ± 0.089 mmHg) after PDA closure compared with the baseline (*P* > 0.05). Also, there was no significant change in the mid and mid-to-apical IVPD and IVPG. Regarding the effect size, the differences in the observed data (Pre- and Post-PDA) were clinically relevant and showed association with a large effect size on total IVPD (rc = 1.0), basal IVPD (rc = 0.889), total IVPG (rc = 0.944), and basal IVPG (rc = 1.0).

### Correlation Between IVPD/IVPG Indices and Echocardiographic Measurements

The correlation results are presented in Table 3. Total IVPD was correlated positively with Ev (*r* = 0.470, *P* = 0.021). Basal IVPD was trended positive with E/Em free wall (*r* = 0.450, *P* = 0.071). Mid-to-apical IVPD was correlated with ESV (*r* = 0.422, *P* = 0.040) and Ev (*r* = 0.424, *P* = 0.040). Apical IVPD was positively correlated with ESV (*r* = 0.677, *P* = 0.001) and negatively with FS (*r* = −0.502, *P* = 0.017). Basal IVPG was correlated with LA/Ao (*r* = 0.446, *p* = 0.033). Mid-to-apical IVPG was correlated with LVIDd (*r* = 0.418, *P* = 0.042), EDV (*r* = 0.434, *P* = 0.034), and ESV (*r* = 0.541, *P* = 0.032). Apical IVPG was correlated with Em of the free wall (*r* = 0.454, *P* = 0.026). Em/Am free wall was negatively correlated with basal IVPD and basal IVPG, and was positively correlated with mid-to-apical IVPD, apical IVPD, mid-to-apical IVPG, and apical IVPG. Neither body weight nor HR was significantly correlated with CMME indices.

**Table 3:** Correlation between CMME indices and echocardiographic measurements.

**Variables**	**HR**	**BW**	**LVIDd**	**EDV**	**ESV**	**FS%**	**LA/Ao**	**Ev**	**Em FW**	**Em/Am FW**	**E/Em FW**
Total IVPD	0.007	0.095	0.211	0.192	0.193	−0.087	0.140	0.470^*^	−0.024	0.148	0.462
Basal IVPD	−0.115	−0.337	0.003	−0.017	−0.251	0.219	0.237	0.131	−0.192	−0.392^*^	0.450
Mid-to-apical IVPD	0.261	0.059	0.272	0.267	0.422^*^	−0.270	−0.219	0.424^*^	0.157	0.458^*^	0.225
Mid IVPD	0.051	−0.089	0.177	0.162	0.044	0.026	−0.163	0.213	−0.149	0.146	0.380
Apical IVPD	0.312	0.024	0.241	0.257	0.677^*^	−0.502^*^	−0.204	0.345	0.426^*^	0.533^*^	−0.161
Total IVPG	−0.263	−0.160	0.334	0.334	0.166	0.055	0.288	0.241	−0.038	−0.247	0.263
Basal IVPG	−0.290	−0.272	0.196	0.185	−0.179	0.307	0.446^*^	0.044	−0.089	−0.489^*^	0.108
Mid-to-apical IVPG	0.121	−0.053	0.418^*^	0.434^*^	0.541^*^	−0.248	−0.080	0.258	0.314	0.434^*^	−0.087
Mid IVPG	0.028	−0.148	0.317	0.319	0.119	0.086	−0.016	0.182	0.187	0.190	−0.010
Apical IVPG	0.215	0.047	0.351	0.370	0.703^*^	−0.420^*^	−0.074	0.281	0.454^*^	0.473^*^	−0.254

*Spearman's correlation between conventional echocardiographic parameters and color M-mode echocardiographic indices*.

* *indicates significant correlation (P < 0.05)*.

## Discussion

In congenital heart disease like PDA, it is important to consider diastolic function and preload separately to understand the pathophysiology and to formulate a treatment plan (32, 33). To date, IVPD and IVPG evaluation *via* CMME is still at the research stage, and the widespread use of such novel methods requires intensive experimental and clinical trials (7, 12, 15). Recent studies have begun to reveal the efficiency of CMME-derived IVPD and IVPG indices for the evaluation of cardiac function. However, this is the first clinical study that explored the short-term changes in IVPD and IVPG in dogs before and rapidly after PDA occlusion.

Regarding conventional echocardiographic parameters, PDA occlusion resulted in a significant reduction of LVIDd, HR, EDV, FS, Ev, Av, Sm septum, and E/Em free wall, as well as a mild non-significant decline in Em and E/Em at the interventricular septum. These changes have been previously reported following PDA closure, and temporal systolic dysfunction may occur in some dogs which seems to be clinically unimportant (5, 6, 34). Based on Frank-Starling's Law, the contractility of the LV is increased secondary to elevated preload through the existing shunt *via* stretching of the muscle fibers to maintain effective systemic circulation. Therefore, reduction of the loading condition of the LV after PDA closure reduces myofibrillar stretching and contractility. In human PDA cases, a severe reduction in the preoperative FS has been reported to be a risk factor for postoperative death (35). Unlikely, postoperative mortalities are less encountered in dogs diagnosed with PDA, as they are usually admitted at <1 year of age before any significant reduction in the FS%. In this study, the FS% was >35% (median of 41.9%), except for one case in which the FS was 28.0%, indicating no reduction of preoperative FS%. The reduction of the Em (for the septum) could be explained by the fact that it can be affected by HR and preload (36). In addition, Em velocity may not reflect the function of the entire ventricle, as it is highly angle-dependent and also vastly influenced by the cardiac translational movement and tethering (17).

Color M-mode echocardiography can be used to separately evaluate the diastolic function and cardiac preload. The feasibility of CMME for the assessment of IVPD and IVPG has already been reported in dogs (11, 12). As in previous studies, IVPD and IVPG were calculated using a program in MATLAB, which was further modified from previous reports (14, 16, 26–28). To enable a detailed evaluation of the pathophysiology of each fraction of IVPD and IVPG in the LV, the LV was divided into three sections according to its anatomical localization, which collectively or partially reflects specific LV function, which implies that IVPD and IVPG can be interpreted comprehensively (26). In this study, CMME was feasible in all dogs and showed moderate variability (95% CV <25%) in all indices except for the apical IVPD and apical IVPG. Those results could be attributed to variable loading conditions that can be influenced by different factors in dogs including age, body weight, and breed of dogs (11). Since all the echocardiographic data were collected without prior sedation, body movements may have resulted in data variability. The higher variability in the apical segment was previously reported (37), which could be explained by variation in the color resolution of the upper part of CMME images compared with the lower part.

In the current study, there was a significant decrease in total and basal IVPD and IVPG post-PDA compared with pre-ligation measurements. This is presumably due to the reduction in the loading status secondary to decreased preload in the dogs post-PDA (9). This explanation is supported by the observed reduction in LVIDd, Ev, and E/Em, which are known as conventional indicators of the preload (25). The apical segment of IVPD and IVPG, on the other hand, increased slightly but not significantly in our study. Additionally, there were no significant changes in mid and mid-to-apical IVPD or IVPG. Generally, PDA closure showed a synchronous decrease in the LV preload and increment in the afterload. The increase in afterload is related to the low-resistance pulmonary circulation compared to the LV outflow (34, 38, 39). A temporal reduction in heart function following PDA ligation was previously reported (5). We assumed a decrease in mid and/or mid-to-apical segments of LV pressure obtained *via* CMME as an indication of LV active relaxation after PDA closure (7). However, this was not proved in our study, which may be due to the small size group, absence of CHF in our patients, and lack of long-term follow-up by CMME. In other words, 48 h was insufficient to observe significant changes in LV active relaxation by CMME. A previous human study required long-term follow-up for recovery of cardiac function after PDA closure (38). In addition, the recovery of diastolic function after PDA closure is slow and difficult to assess (40). Therefore, long-term observations of IVPD and IVPG may allow better assessment of changes in LV mitigation capacity. Based on our results, we believe that left ventricular relaxation capacity is less susceptible to sudden hemodynamic changes that may be detectable by CMME over a longer time of patient follow-up.

In our study, there were some correlations between the CMME-derived indices and conventional echocardiographic parameters, particularly those that are important for diastolic function, as previously observed in other studies (7, 9–12, 16, 37). The IVPD and IVPG indices reported in the present study were comparable to previous reports in dogs and cats (11, 15, 37). A previous study reported CMME-derived IVPD and IVPG indices in a group of healthy small to large breed dogs of different ages and BW and found that the IVPD was influenced by the heart size (11). In our study, there was a significant correlation between mid-to-apical IVPG and LVIDd, which could be attributed to the narrow age and BW ranges of our group. Another feline study showed that the total IVPG was affected by HR (37), which was not found in our study. There was not much difference in the HR range between dogs in this study and cats in the other study (37). Unlike dogs, cats can become highly excited during the examination, and consequent fluctuation of the HR and increased systolic function may be noticed under the influence of a high sympathetic tone (37, 41). In our study, the correlation coefficient was almost weak. Therefore, proper interpretation of these relationships may be biased due to the small sample size and short-term follow-up, and we would like to accumulate a larger number of PDA cases throughout a longitudinal observation to increase the statistical power of our findings in the future.

### Clinical Implications

This is the first clinical study to utilize CMME indices in dogs to compare the changes observed before and after PDA occlusion. The clinical relevance of the result is clear and has been associated with a large effect size on total and basal segments of IVPD and IVPG rather than mid-to-apical parts. The result of this study did not provide clear evidence of significant change in mid-to-apical IVPD/IVPG, the landmark for enhanced LV relaxation, because assessment after 48 h following PDA occlusion is too short to fully restore left ventricular diastolic function. This is likely due to the more rapid changes in hemodynamic rather than morphological changes in the cardiac function shortly after the PDA occlusion, and further longitudinal observation at different time points is necessary.

### Limitations

The number of dogs included in the study is small; however, the provided data and their clinical relevance are quite solid. Since the PDA operation was performed *via* intercostal thoracotomy, neither catheterization nor angiography was performed for this study. A previous study showed that CMME-derived IVPD and IVPG were strongly correlated with *tau* measured by invasive catheterization, thus comparative assessment of IVPD between CMME and catheterization was not performed in this study (7, 12, 28). Besides, recent publications used CMME for non-invasive assessment of IVPD and/or IVPG that has been performed in dogs and cats without catheterization (15, 37).

## Conclusion

A short-term observation of IVPD and IVPG reflects the changes in preload rather than myocardial relaxation, and a continuous follow-up at different time intervals following PDA occlusion warrants another study. Overall, CMME-derived IVPD and IVPG can be used for further evaluation of cardiac function in dogs, and the apical indices should be considered with caution.

## Data Availability Statement

The raw data supporting the conclusions of this article will be made available by the authors, without undue reservation.

## Ethics Statement

The animal study was reviewed and approved by the Ethical Committee of the Animal Medical Center, Tokyo University of Agriculture and Technology. Written informed consent was obtained from the owners for the participation of their animals in this study.

## Author Contributions

MH, SG, and RT conceived and designed the experiment. RT, SG, and KS: surgical procedures. MH, ASM, KM, LH, KT, and TY: echocardiography and software analysis. MH and ASM: statistical analysis, manuscript drafting, and writing the final manuscript. LH, AU, KS, KT, and RT revised and edited the manuscript. All authors have read and agreed to the published version of the manuscript.

## Conflict of Interest

The authors declare that the research was conducted in the absence of any commercial or financial relationships that could be construed as a potential conflict of interest. The handling editor HS declared a past co-authorship with the authors ASM and RT.

## Publisher's Note

All claims expressed in this article are solely those of the authors and do not necessarily represent those of their affiliated organizations, or those of the publisher, the editors and the reviewers. Any product that may be evaluated in this article, or claim that may be made by its manufacturer, is not guaranteed or endorsed by the publisher.
